# Metabolic Reprogramming of Vascular Endothelial Cells: Basic Research and Clinical Applications

**DOI:** 10.3389/fcell.2021.626047

**Published:** 2021-02-18

**Authors:** Hanlin Peng, Xiuli Wang, Junbao Du, Qinghua Cui, Yaqian Huang, Hongfang Jin

**Affiliations:** ^1^Department of Pediatrics, Peking University First Hospital, Beijing, China; ^2^Key Laboratory of Molecular Cardiovascular Sciences, Ministry of Education, Beijing, China; ^3^Department of Biomedical Informatics, Centre for Non-coding RNA Medicine, School of Basic Medical Sciences, Peking University, Beijing, China

**Keywords:** energy metabolism, metabolic reprogramming, endothelial cell dysfunction, glycolysis, mitochondrial oxidation

## Abstract

Vascular endothelial cells (VECs) build a barrier separating the blood from the vascular wall. The vascular endothelium is the largest endocrine organ, and is well-known for its crucial role in the regulation of vascular function. The initial response to endothelial cell injury can lead to the activation of VECs. However, excessive activation leads to metabolic pathway disruption, VEC dysfunction, and angiogenesis. The pathways related to VEC metabolic reprogramming recently have been considered as key modulators of VEC function in processes such as angiogenesis, inflammation, and barrier maintenance. In this review, we focus on the changes of VEC metabolism under physiological and pathophysiological conditions.

Vascular endothelium has a variety of physiological functions, for instance, serving as a barrier between blood and tissues and acting as an endocrine organ in the body (Krüger-Genge et al., 2019). Indeed, vascular endothelial cells (VECs) can synthesize and release various endothelial-derived vasoactive factors to regulate blood flow, coagulation, and fibrinolytic balance. VEC activation is defined as stimulus-induced quantitative changes in the expression of specific genes (Pober, 1988), and excessive activation causes endothelial cell dysfunction or unnecessary angiogenesis. VEC activation and dysfunction are characteristics of atherosclerosis, diabetes, obesity, and aging (Theodorou and Boon, 2018). And in the development of atherosclerosis, VEC activation and dysfunction in vulnerable areas of arterial vessels are among the earliest changes that can be detected (Davignon and Ganz, 2004). Moreover, tumor neovascularization is characterized by a highly disordered vascular network and the generation of new blood vessels. Despite in-depth studies of cellular metabolism, the metabolic properties of VECs have only recently been a focus of research (Eelen et al., 2018). It is found that changes in VEC metabolism lead to endothelial cell dysfunction and are closely related with the pathogenesis of many diseases such as atherosclerosis, diabetic angiopathy, pulmonary hypertension, etc. (Pober et al., 2009). Based on these findings, VEC metabolism is becoming a new therapeutic target for various diseases. Nevertheless, the relationship between VEC metabolism and the pathogenesis of vascular dysfunction and remodeling remains incompletely understood. Overall, in this review, we briefly summarize the current understanding of the metabolic changes in dysfunctional VECs, provide a detailed overview of the metabolic pathways activated under normal and pathological conditions, in order to identify potential therapeutic targets on the VEC metabolic reprogramming.

## VEC Metabolism Under Normal Physiological Condition

Theoretically, since VECs are exposed to oxygen from the bloodstream, they are expected to produce adenosine triphosphate (ATP) through oxidative phosphorylation (OXPHOS) for energy-yielding. Nevertheless, due to the relatively low mitochondrial content of VECs, energy production is still mainly dependent of glycolysis which yields more than 85% of ATP under normal physiological condition (De Bock et al., 2013b). Conversely, glucose-derived pyruvate enters the mitochondria to participate in the metabolism of glucose via the tricarboxylic acid cycle (TCA), resulting in the production of ATP, which is less than 1% (De Bock et al., 2013b). Although the glycolysis-derived ATP by per mole glucose is low, ATP generated from glycolysis in a short time period is more quickly than that from OXPHOS in the presence of an unrestricted amount of glucose (Eelen et al., 2015), because VECs have a higher glycolytic rate than many other healthy cell types (De Bock et al., 2013b). VEC aerobic glycolysis also has the following advantages over other processes: (1) a reduction of reactive oxygen species (ROS) produced by OXPHOS; (2) the ability to maximize oxygen transfer to cells surrounding the blood vessel; (3) the ability to adapt to hypoxic environments, and (4) the production of lactate, which can promote angiogenesis (Eelen et al., 2015).

Another advantage of glycolysis is the possible shunt of glucose to side branches, including the hexosamine biosynthesis pathway (HBP), pentose phosphate pathway (PPP), and polyol pathway (PP) for biomacromolecule synthesis. Of all the glucose utilized by VECs, only 1–3% enters the PPP under physiological condition (De Bock et al., 2013a). The PPP leads to the generation of nicotinamide adenine dinucleotide phosphate (NADPH) and ribose-5-phosphate (R5P). NADPH can convert oxidized glutathione (GSSG) to glutathione (GSH) and maintain the internal redox balance. And R5P can be used to synthesize nucleotides (De Bock et al., 2013a). On the other hand, although the role of the HBP in VECs *in vivo* is not clear, *N-*acetylglucosamine produced by this pathway is an essential substance for *N-*glycosylation and *O-*glycosylation and may be the key to the glycosylation of angiogenic proteins (Luo et al., 2008). Finally, when the amount of glucose exceeds the capacity of glycolysis, glucose enters the PP, where glucose is catalyzed to sorbitol by aldose reductase (AR), and later is converted to fructose. Since AR requires NADPH to provide reducing power, PP activation consumes NADPH, thus leading to the accumulation of ROS (De Bock et al., 2013a).

In addition to glucose, fatty acids (FAs) are another energy source for VECs. FAs can be converted into acetyl-CoA, and the latter can be used to generate reducing power and produce ATP in the mitochondria. However, the VEC mitochondria serve as a signaling switching station such as mitochondrial calcium signaling, ROS generation from electron transport chain and NO production catalyzed by eNOS, rather than power factories (Davidson, 2010; Groschner et al., 2012). Therefore, fatty acid oxidation (FAO) occurring in the mitochondria may not contribute substantially to total ATP production in VECs. Though mitochondrial is not that important for providing energy, the disturbed mitochondrial dynamics contributes to VEC dysfunction and the development of vascular diseases (Tang et al., 2014). Kalucka et al. found that FAO in the silence ECs is important for maintaining redox homeostasis and EC functions (Kalucka et al., 2018), while FAO in proliferative ECs is also indispensable for *de novo* dNTP synthesis (Schoors et al., 2015).

To date, the metabolism of amino acid in VECs is not well studied except for arginine and glutamine metabolism. Nitric oxide (NO), a crucial modulator of VEC function, is generated from arginine by endothelial nitric oxide synthase (eNOS) (Tousoulis et al., 2012). Interestingly, the conversion of glutamine into glucosamine inhibits the activity of the oxidative PPP (oxPPP), thereby leading to the reduction of NADPH (an important cofactor for eNOS) and inhibiting the production of endothelial NO (Wu et al., 2001). Moreover, glutamine-derived glutamate can be the substrate to be used in the other non-essential amino acids production. It also acts as a supplementary carbon source in the TCA cycle for ATP production after further metabolized into α-ketoglutarate (Kim B. et al., 2017).

**FIGURE 1:**
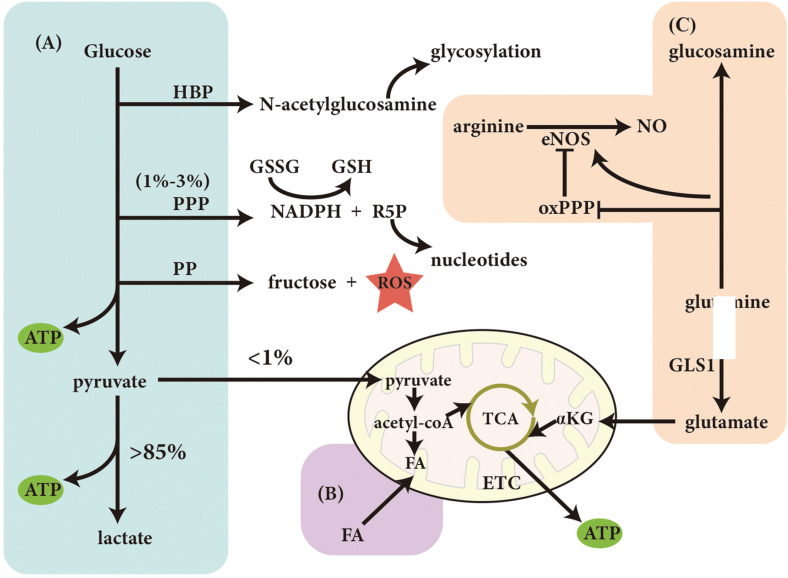
Vascular endothelial cell metabolism under normal physiological conditions. Metabolism of **(A)** glucose, **(B)** fatty acid, **(C)** amino acid. ATP, adenosine triphosphate; HBP, hexosamine biosynthesis pathway; PPP, pentose phosphate pathway; PP, polyol pathway; GSH, glutathione; GSSG, oxidized glutathione; NADPH, nicotinamide adenine dinucleotide phosphate; R5P, ribose-5-phosphate; ETC, electron transport chain; TCA, tricarboxylic acid cycle; FA, fatty acid; eNOS, endothelial nitric oxide synthase; oxPPP, oxidized PPP; GLS1, glutaminase 1, αKG: α-ketoglutarate.

In summary, the main energy sources of VECs is derived from glucose glycolysis, whereas FAO and glutamine oxidation are generally thought to supplement the TCA cycle via OXPHOS (Figure 1). When the rate of glycolysis decreases, the energy provided by the oxidative metabolism of glucose, FAs, and amino acids might alternatively increase for supporting the VEC activity (Krutzfeldt et al., 1990).

## Metabolic Reprogramming in Dysfunctional VECS

Many factors can activate VECs, such as lipopolysaccharide (LPS), interleukin 1 (IL-1) and tumor necrosis factor (TNF-α) (Magnuson et al., 1989). After activation, VEC metabolism is disordered, represented by the increased glycolysis, and increased expression level and activity of fatty acid synthase (FAS) (Pan et al., 2009; De Bock et al., 2013b; Feng et al., 2017; Singh et al., 2017; Li et al., 2019b). These enhance the proliferation, migration and inflammation of VECs, leading to VEC dysfunction and vascular diseases. In the next paragraphs, we will review in detail the metabolic changes and VEC dysfunction.

### Metabolic Reprogramming and VEC Migration

VECs migrate to an anoxic microenvironment in the tumor angiogenesis development. The energy supporting the above process is mainly derived from VEC anaerobic metabolism. Therefore, the migrated VECs are highly dependent of glycolysis compared with silence VECs. In VECs, glycolysis normally occurs in the perinuclear cytoplasm; however, once these cells begin to migrate, glycolysis also takes place in lamellipodia and filopodia to promote the rapid production of ATP needed for migration (De Bock et al., 2013b). Glucose-6-phosphate dehydrogenase (G6PD) is the rate-limiting enzyme of PPP, and the overexpression of G6PD will stimulate VEC migration due to the increase in NO and NADPH production (Pan et al., 2009). Conversely, an increased glucosamine concentration leads to protein glycosylation and inhibits the migration of VECs (Li et al., 2019b).

### Metabolic Reprogramming and VEC Inflammation

The metabolic changes of inflammatory VECs are mainly characterized by increased glycolysis. The study showed that mechanical low shear stress activated hypoxia-inducible factor 1α (HIF-1α) in cultured VECs via activating the nuclear factor-κB (NF-κB) pathway and promoting the expression of deubiquitinating enzyme cezanne. HIF-1α promotes the production of inflammatory factors in VECs by increasing the expression of the glycolysis-related regulators hexokinase 2 (HK2), enolase 2 (ENO2), glucose transporter 1 (GLUT1), fructose-2,6-biphosphatase 3 (PFKFB3) and extracellular acidification rate (ECAR, a direct marker of glycolysis) (Feng et al., 2017).

### Metabolic Reprogramming and VEC Proliferation

Studies have found that multiple steps in FA metabolism, such as FA synthesis, FA transport and FAO, are all involved in the proliferation of VECs. Firstly, the expression and activity of FAS are increased in hypoxic human pulmonary artery endothelial cells (HPAECs), while the inhibition of FAS leads to the reduced HPAEC proliferation (Singh et al., 2017). Secondly, VEC proliferation is associated with the expression of fatty acid transporters (FATPs) and fatty acid binding proteins (FABPs). For instance, the silencing of *FABP4* inhibited the VEC proliferation *in vitro* (Elmasri et al., 2012). Also, the downregulation of carnitine palmitoyl transferase 1A (CPT1A) expression, a rate-limiting enzyme in the metabolism of FA, or *CPT1A* knockout was also found to inhibit the proliferation of VECs (Schoors et al., 2015). The above results indicate that fatty acid metabolism plays a significant role in controlling VEC proliferation. However, the detailed mechanisms need further studies.

The proliferation of VECs also depends on glutamine metabolism. Indeed, genetic deletion or pharmacological inhibition of glutaminase1(GLS1) inhibits the proliferation of VECs (Kim B. et al., 2017). Asparagine synthetase (ASNS) is an enzyme which is responsible for the generation of asparagine from nitrogen (glutamine-derived) and aspartic acid. Huang et al. found that the silencing of *ASNS* inhibited the proliferation of VECs (Huang et al., 2017).

## Molecular Mechanisms and Signaling Pathways Involved in the Regulation of VEC Metabolism

**FIGURE 2:**
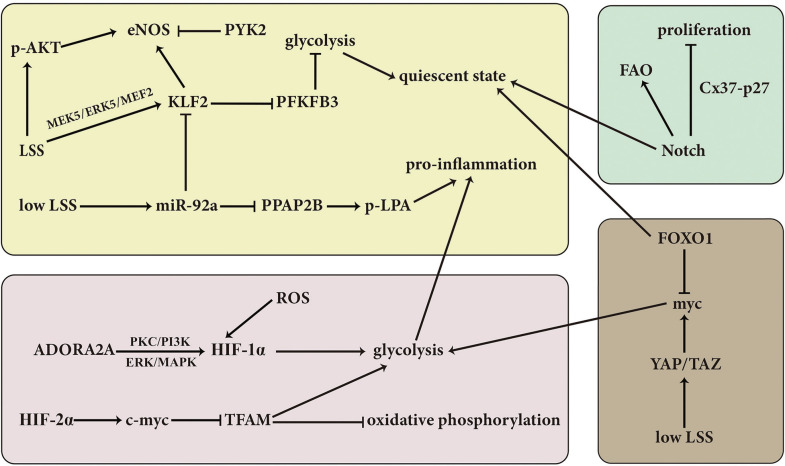
Overview of the molecular mechanisms and signaling pathways involved in the regulation of VEC. LSS, laminar shear stress; KLF2, Kruppel-like factor 2; PFKFB3, fructose-2,6-biphosphatase 3; PPAP2B, phosphatidic acid phosphatase type 2B; LPA, lysophosphatidic acid; AKT, protein kinase B; eNOS, endothelial nitric oxide synthase; PYK2, proline-rich tyrosine kinase 2; ROS, reactive oxygen species; HIF-1α, hypoxia inducible factor-1α; PKC, protein kinase C; ADORA2A, adenosine A2a receptor; FOXO1, forkhead box O1; FAO, fatty acid oxidation; YAP, Yes-associated protein; TAZ, transcriptional coactivator with PDZ-binding motif; p, phosphorylation.

Various signaling pathways are involved in the regulation of VEC metabolism to respond quickly to changes under environmental conditions (Figure 2; Li et al., 2019c).

Laminar shear stress (LSS) is mainly transduced via the activation of a mechanical signal transduction pathway triggering a certain response of VECs to the external environment; at the same time, LSS can also affect VEC metabolism. It is reported that the expression of transcriptional factor Kruppel like factor 2 (KLF2) is induced in the LSS-treated VECs via the activation of MEK5/ERK5/MEF2 signaling pathway (Parmar et al., 2006). Subsequently, a great number of genes controlling endothelial energy metabolism, thrombosis/hemostasis, inflammation, vascular tone, and vessel development/remodeling are regulated by the LSS-induced KLF2 pathway (SenBanerjee et al., 2004; Parmar et al., 2006). For example, PFKFB3 is a key glycolytic enzyme. It was found that LSS-upregulated KLF2 reduced glucose uptake and glycolysis by inhibiting the activity of the *PFKFB3* promoter, then decreased the flux of glycolysis and maintained VECs at a resting state (Dekker et al., 2002; Doddaballapur et al., 2015). On the other hand, under normal condition, phosphatidic acid phosphatase type 2B (PPAP2B) dephosphorylates lysophosphatidic acid (LPA) to prevent it from binding to its receptor LPAR1 and then inhibits VEC inflammation. While, low LSS leads to the increased miR-92a and decreased KLF2, which inhibits the expression of PPAP2B in VECs, and then induces the VEC inflammation (Wu et al., 2011, 2015). Another key point in the response to LSS is the activity of eNOS. ECs possess special membrane organelle cilia, which is responsible for sensing the LSS and thus controlling the production of NO (Nauli et al., 2008). However, the underlying mechanisms by which LSS regulates the activity of eNOS are quite sophisticated. For example, LSS-induced phosphorylation of AKT and increased expression of KLF2 can activate and promote the expression of eNOS, respectively, but upregulation of proline-rich tyrosine kinase 2(PYK2) leads to opposite result (Zhou et al., 2014).

Like KLF2, SIRT3 also regulates the metabolic shift between mitochondrial oxidation and glycolysis, targeting on the PFKFB3. It was found that SIRT3 KO-EC exhibited a reduction of glycolysis and an elevation in mitochondrial oxidation and ROS formation, accompanying with the downregulated expression of PFKFB3 and upregulated acetylation of PFKFB3. The abovementioned EC metabolic shift was regarded to contribute to an impaired angiogenesis, reduced coronary flow reserve and diastolic dysfunction in SIRT3 KO mice (He et al., 2017).

Some studies have suggested that hypoxia signaling also participates in the regulation of VEC metabolism. For example, the upregulation of pyruvate dehydrogenase kinase 1(PDK1) induced by HIF-1 in the disturbed flow-activated ECs results in the phosphorylation and inactivation of pyruvate dehydrogenase (PDH), then blocks the conversion from pyruvate to acetyl-CoA catalyzed by PDH, and finally suppresses the TCA cycle. At the same time, the increased glucose transporter and glycolytic enzyme genes such as GLUT1, lactate dehydrogenase A (LDHA) and HK-2 due to the activation of HIF-1 pathway are also involved in the disturbed flow-activated EC metabolic reprogramming and inflammation (Prabhakar and Semenza, 2012; Wu et al., 2017). Moreover, in hypoxic pulmonary artery ECs, HIF-2α inhibits mitochondrial transcription factor A (TFAM) by downregulating the expression of c-myc, and thus leads to the suppression of mitochondrial gene expression and the decrease in oxidative phosphorylation (Zarrabi et al., 2017). Regarding the mechanism responsible for the activation of HIF signaling, NAD(P)H Oxidase-4 (NOX4)-derived ROS is found to be required for the disturbed flow-activated HIF-1 pathway and subsequently increased glycolysis and decreased mitochondrial respiration (Wu et al., 2017). Besides, protein kinase C (PKC) and PI3K signaling are also involved in the hypoxia-induced HIF-1α activation and reinforced glycolysis in hypoxic VECs (Paik et al., 2017). Except for the above signaling pathways, adenosine A2a receptor (ADORA2A) in hypoxic VECs can enhance HIF-1α protein synthesis via ERK/MAPK and PI3K/Akt pathways and therefore promote glycolytic enzyme expression, glycolytic flux, EC proliferation, and angiogenesis (Liu et al., 2017).

The activation of Notch signaling pathway is necessary for maintaining the quiescent state of VECs. Kalucka et al. found that the treatment with Notch pathway stimulator Dll4 in the proliferating ECs induced a significant quiescence of ECs represented by the fact that the treated ECs became less proliferative and more in G0 phase. Simultaneously, the transcriptome results showed that the expression of genes related with glycolysis, TCA cycle, nucleotide synthesis, and purine/pyrimidine synthesis were decreased while the expression of genes controlling FAO was increased (Kalucka et al., 2018). Furthermore, it was found that Notch signaling led to the increased FAO flux, which was utilized for redox homeostasis through the generation of NADPH in quiescent VECs. The abovementioned effect of Notch signaling was mediated by the upregulation of CPTA1 (Kalucka et al., 2018). Moreover, Notch signaling is activated in the fluid shear stress-induced EC quiescence model, in which Cx37-p27 serve as the downstream of Notch signaling to promote the EC cell cycle arrest (Fang et al., 2017).

Similarly, forkhead box O1 (FOXO1) also is an important switch bridging the EC growth status and metabolic activities (Wilhelm et al., 2016). FOXO1 is found to decrease glycolysis, reduce mitochondrial respiration, inhibit EC proliferation and sprouting angiogenesis, and thereby maintain the EC in a quiescence status. EC-specific deletion of FOXO1, however, results in an uncontrolled EC proliferation, vessel hyperplasia and enlargement. Furthermore, it is found that the constitutive activation of FOXO1 reduces the myc expression, promotes myc degradation, and increases the expression of the negative regulators of myc MXI1. Considering that myc is a strong driver of glycolysis, mitochondrial metabolism, and cell growth, the antagonization of myc might mediate the pro-quiescence effect of FOXO1. FOXO1/myc acts a novel metabolic gatekeeper for switching the EC proliferation to quiescence (Wilhelm et al., 2016). On the contrary, endothelial Yes-associated protein/transcriptional coactivator with PDZ-binding motif complex (YAP/TAZ) promotes the formation and maturation of brain vessel in mice by upregulating myc-drive glycolysis, mitochondrial oxidative phosphorylation and EC proliferation (Kim J. et al., 2017). Low LSS activates YAP/TAZ by enhancing the activity of c-Jun N-terminal kinase (Wang et al., 2016), and the activation of integrin α5β1 and c-Abl induced by low LSS can upregulate the nuclear translocation of YAP by phosphorylating YAP at Y357 (Li et al., 2019a). Additionally, the RAF-MEK-ERK pathway activates glutaminolysis in VECs to support the metabolic requirements of highly proliferative VECs (Guo et al., 2016).

## VEC Metabolism Under Pathological Condition

**FIGURE 3:**
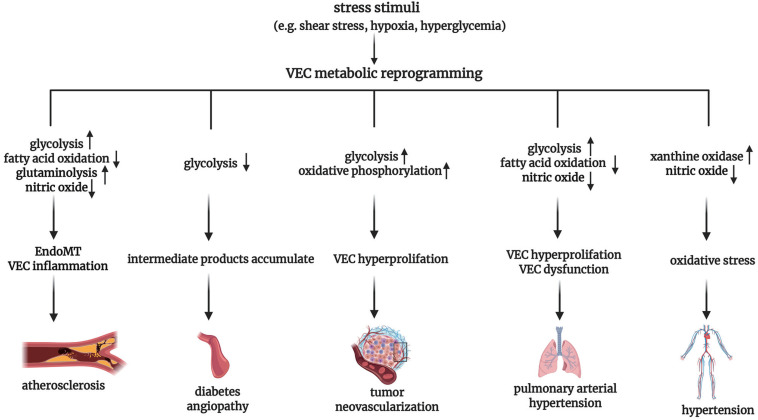
Overview of vascular endothelial cell metabolic reprogramming in the pathogenesis of vascular diseases. VEC, vascular endothelial cell; EndoMT, endothelial-to-mesenchymal transition.

Under normal physiological conditions, the main energy source of VECs comes from glycolysis. However, the increased activity of glycolytic-related enzymes after VEC activation can trigger the pro-inflammatory pathway, contributing to the progress of vascular injury diseases like atherosclerosis. Conversely, when the activity of glycolysis-related enzymes is reduced, resulting in stagnated glycolysis, intermediate products accumulate and can be metabolized via other pathways. The consequent generation of a great number of oxidative substances, the generation of advanced glycation end product (AGE), and eNOS uncoupling can lead to diabetic angiopathy. Furthermore, VECs frequently show a high glycolytic phenotype while retaining functional mitochondria during tumor neovascularization. Finally, metabolic reprogramming of VECs is also involved in the pathogenesis of hypertension and pulmonary arterial hypertension (Figure 3). In the following paragraphs, we shall review in further detail the abnormal VEC metabolism and its significance under pathophysiological conditions.

### VEC Metabolism in Atherosclerosis

One of the most crucial factors for the initiation of VEC activation is disturbed blood flow dynamics (Davignon and Ganz, 2004). The disturbed blood flow mainly leads to the metabolic changes of VEC glycolysis. As mentioned in the above text, the expression of transcription factor KLF2 is upregulated by high LSS, resulting in decreased glycolysis and mitochondrial respiration by inhibiting the promoter of *PFKFB3* (Doddaballapur et al., 2015). As a result, KLF2 is thought to be a key transcriptional switch point regulating the quiescent and activated states of VECs. On the other hand, VECs in the atherosclerotic region of the vascular system are disrupted by low LSS. Under low LSS, the activation of NF-κB induces elevated HIF-1α mRNA and increased the expression of cezanne, a ubiquitin-editing enzyme preventing the HIF-1α protein degradation, thus stabilizing HIF-1α. And then, HIF-1α promotes the proliferation and inflammation of VECs by activating glycolytic genes HK2, ENO2, and PFKFB3 to upregulate glycolysis, resulting in the initiation of atherosclerosis (Feng et al., 2017).

Endothelial-to-mesenchymal transition (EndoMT) is an important pathological basis of atherosclerotic lesions. These VEC-derived mesenchymal cells can cause the instability of plaque by enhancing the expression and activity of collagen-matrix metalloproteinase (Evrard et al., 2016). Xiong et al. demonstrated a metabolic mechanism responsible for EndoMT targeting on the FAO-controlled acetyl-CoA. It was found that the induction of EndoMT was associated with a reduction in FAO demonstrated by a decrease in acetyl-CoA levels and a fall in the expression of CPT1A, the enzyme that played rate-limiting and obligate roles in FAO. The downregulation of CPT1A expression in ECs promoted the EndoMT, while the increase in acetyl-CoA levels by supplementing acetate inhibited the SMAD2 activation and EndoMT program in cytokine-stimulated ECs. Those abovementioned results suggest the causal relationship between the reduction of FAO in the ECs and EndoMT, and provide a novel metabolic therapeutic choice for EndoMT-related diseases such as atherosclerosis (Xiong et al., 2018).

It has been proved that the pro-inflammatory YAP/TAZ signaling promotes the glutaminolysis of VECs, suggesting that glutamine has an atherogenic effect (Bertero et al., 2016). Glutamine deficiency can lead to the increased endoplasmic reticulum stress, impaired TCA, inhibition of mTOR signaling and subsequently reduced protein synthesis (Huang et al., 2017). Moreover, glutamate can be converted to neurotransmitter gamma aminobutyric acid (GABA), an anti-inflammatory mediator, in HAECs and HUVECs, and therefore glutamine deficiency may reduce the GABA level and its anti-inflammatory effect (Sen et al., 2016). In atherosclerosis, VECs also show disruption in metabolic pathways related to NO production. In the endothelium, NO-mediated vasodilation is required for vascular homeostasis and the inhibition of important events that promote atherosclerosis, including platelet aggregation, and smooth muscle cells migration (Kawashima, 2004). Consistently, one of the early features of atherosclerosis is eNOS uncoupling, resulting in an imbalance between anti-atherosclerotic NO and pro-atherosclerotic peroxides (Pircher et al., 2016).

### VEC Metabolism in Diabetic Angiopathy

As a key metabolic characteristic of diabetes, hyperglycemia is found to be closely correlated with the alterations of VEC metabolism, VEC dysfunction and consequent diabetic angiopathy. A significantly increased production of ROS and reactive nitrogen species (RNS) is an important change of diabetic VEC metabolism (Goveia et al., 2014). Hyperglycemia leads to an increased ROS level in VEC mainly by three mechanisms: (1) an obviously increased expression and activity of NAD(P)H oxidase protein subunits (p22phox, p67phox, and p47phox), which was observed in the veins and arteries of diabetic patients (Guzik et al., 2002). The NADPH-derived ROS production is associated with the downregulation of 8-oxoguanine glycosylase and subsequent activation of PKC in high glucose-treated HUVECs (Xie et al., 2020); (2) xanthine oxidase (XO) inhibitor allopurinol can prevent hyperglycemia-induced generation of ROS, which indicates that XO may be the major contributor of ROS (Eleftheriadis et al., 2018); (3) uncoupled eNOS can induce the increase of oxidative stress in diabetic mice (Sasaki et al., 2008). Besides, hyperglycemia results in an increase in cAMP, and then through the cAMP-dependent PKA, G6PD is phosphorylated and its activity is inhibited. As a result, the entry of glucose into PPP is reduced, leading to the subsequent decreased NADPH and increased ROS (Zhang et al., 2000). Moreover, when exposed to high glucose, the expression of fission proteins fission-1 (Fis1) and dynamin-related protein-1 (Drp1) in the VEC mitochondrial is enhanced and leads to the increase in the mitochondrial fission and an impaired autophagy of mitochondrial. And then the altered mitochondrial dynamics results in an increase of ROS generation, an inhibition of eNOS activation, and the loss of NO bioavailability (Shenouda et al., 2011).

Excess ROS production in diabetic VECs can activate poly-adenosine diphosphate ribose polymerase 1 (PARP-1), which mediates the ribosylation of poly-ADP, thereby inactivating the key glycolytic enzyme glyceraldehyde triphosphate dehydrogenase (GAPDH), whose activity plays a vital role in the maintenance of glycolytic flux (Du et al., 2003). As a result, the inhibited GAPDH leads to the stalled glycolysis. Excess glucose that cannot be metabolized due to the stalled glycolysis enters the PP and is converted to sorbitol at the cost of NADPH by AR, thereby leading to an increase in ROS. Sorbitol is then switched to fructose and highly active 3-deoxyglucose (3DG), promoting the formation of AGEs (Goveia et al., 2014). And the accumulated glycolysis intermediates then turn to three abnormal metabolic pathways as follows. (1) Glycolytic-derived fructose 6-phosphate can produce fructose-6-phosphate (F6P) via 6-phosphate fructosyl amide transferase; and the accumulated F6P is then mainly metabolized through the HBP, which produces the important precursor of glycosylation, uracil-*N*-acetylglucosamine diphosphate (UDP-GlcNAc) (Brownlee, 2001). Although glycosylation is an important process for the physiological function of VECs, hyperglycemic-induced protein glycosylation may inhibit angiogenesis (Luo et al., 2008). (2) The intermediates of glycolysis glyceraldehyde 3-phosphate (G3P) and dihydroxyacetone phosphate (DHAP) are transferred to the methylglyoxal pathway, which further boosts the production of AGEs (Goveia et al., 2014). Moreover, G3P and DHAP contribute to the diacylglycerol *de novo* synthesis, and subsequent PKC activation causes vascular abnormalities (Das Evcimen and King, 2007). (3) In theory, the accumulated glucose-6-phosphate (G6P) can enter the glucuronate cycle, but there is a lack of comprehensive research on the glucuronate cycle in diabetic VECs (Eelen et al., 2018).

### VEC Metabolism in Tumor Neovascularization

The vessels in tumor are highly abnormal, and mainly characterized by VEC hyperproliferation. The switch of VECs from the quiescent state to the proliferative and migratory state during tumor neovascularization is closely related with the VEC metabolic reprogramming. VECs in tumors show a greater dependence on glycolysis to produce ATP than healthy VECs, mainly manifested as increased expression levels of associated genes, such as the glucose transporter *GLUT1* and the glycolytic activator *PFKFB3*, and thus exhibit a high glycolytic phenotype. The upregulation of PFKFB3 in the tumor depends on hypoxia, pro-inflammatory cytokines, or hormone signaling in the microenvironment (Zecchin et al., 2017). In addition, PPP and the serine biosynthetic pathway, which are utilized for the synthesis of nucleotide and biomass, are more highly activated in tumor VECs than in healthy VECs (Rohlenova et al., 2018).

Both tumor and healthy VECs retain functional mitochondria. The active OXPHOS increases the flexibility of using other substrates for energy generation, and provides metabolites for the synthesis of biomass to support cell proliferation. Studies revealed that the death of proliferating tumor VECs caused by mitochondrial respiratory inhibition is more than that of quiescent VECs, which indicated that functional OXPHOS may be quite important for the proliferating tumor VECs (Rohlenova et al., 2018). The role of mitochondria in tumor endothelial cells deserves further study.

### VEC Metabolism in Pulmonary Arterial Hypertension

The high proliferation and dysfunction of VECs are main characteristics of pulmonary arterial hypertension (PAH) (Yu and Chan, 2017). The excessive proliferation of VECs in PAH depends on an increased glycolysis flux and a decreased oxygen consumption related to the upregulation of HIF-1α (Tuder et al., 2012). Moreover, bone morphogenetic protein receptor type 2 (*BMPR2*) is an important human PAH pathogenic gene (Majka et al., 2011). The expression of a BMPR2 mutant protein in human lung VECs contributes to the progress of PAH mainly by subsequent metabolic changes in VECs: (1) upregulated expression of glycolysis-related enzymes; (2) depressed carnitine metabolism and fatty acid oxidation;, and (3) decreased TCA cycle intermediates (Fessel et al., 2012). In addition, the increase in isocitrate dehydrogenase (IDH)-1 and IDH-2 activity observed in *BPMR2*-mutated VECs is identical with the increased serum IDH activity of PAH patients (Fessel et al., 2012).

Another characteristic of PAH is the decrease of NO content in VECs, which may be related to the following two aspects: (1) the decrease in antioxidant manganese superoxide dismutase (MnSOD) in mitochondria since MnSOD increases the biological activity of NO by scavenging the superoxide anion (Fijalkowska et al., 2010); and (2) the high expression level of arginase II, which competes with eNOS for the substrate arginine, thus leading to the subsequent decreased production of NO (Xu et al., 2004).

### VEC Metabolism in Hypertension

In addition to the above EC metabolic changes, the abnormal purine catabolism in VECs is involved in the EC dysfunction in the development of hypertension. Xanthine oxidoreductase (XOR) catalyzes the oxidation of hypoxanthine to xanthine, and then xanthine to uric acid (UA). XOR exists in two different forms: xanthine dehydrogenase (XD) and XO (Maruhashi et al., 2018). The differences between XD and XO include the following aspects: (1) XD is the main enzyme found in normal tissues, but its activity is low, and XO dominates in tissues with injury and ischemia (Schmitz and Brand, 2016). (2) They use different electron acceptors. NAD^+^ is preferentially used in the XD catalyzed-reaction, in which a stable reaction product NADH is generated; while molecular oxygen is preferentially used in the XO catalyzed-reaction, resulting the generation of superoxide anion and hydrogen peroxide (Maruhashi et al., 2018).

Compared with Wistar-Kyoto rat, XO activity was increased in VECs of spontaneously hypertensive rat (SHR), which was related with an elevation of arteriolar tone (Vila et al., 2019). Besides ROS generated from XO catalyzed-reaction, the concomitant product UA is regarded to be closely related with EC dysfunction and hypertension. Correspondingly, UA-lowering drugs have a significant anti-hypertensive effect via protecting EC in the basic experiment and clinical trials (Otani et al., 2018; Yang et al., 2018). The proposed mechanisms responsible for UA-related EC dysfunction involved the followings: (1) the impairment NO production and bioavailability. UA inhibited phosphorylation of eNOS and production of NO via PI3K/Akt pathway (Choi et al., 2014). Also, UA suppressed NO release and reacted directly with NO in an irreversible manner leading to NO depletion (Kang et al., 2005; Gersch et al., 2008). (2) the pro-inflammatory effect. UA treatment in VECs upregulated the mRNA expression, protein and release of inflammatory mediator C-reactive protein via p38 and ERK pathway (Kang et al., 2005). (3) the impaired mitochondrial function. UA-treated human aortic ECs exhibited the reduction mitochondrial mass and ATP production, the decrease in aconitase-2 activity and expression of enoyl CoA hydratase-1 (Sánchez-Lozada et al., 2012). (4) the induction of EC phenotype transition. UA-induced EndoMT in HUVECs via ROS generation and glycocalyx shedding (Ko et al., 2019). (5) The inhibition of EC proliferation and migration (Gersch et al., 2008). However, it is worth noticing that UA is also an antioxidant. Its main properties include scavenging hydroxyl free radicals, superoxide anions, peroxynitrite and preventing lipid peroxidation (Amaro et al., 2019).

## Key Insights and Therapeutic Perspective

In summary, VECs mostly remain quiescent, but can be activated by various physiological and pathological stimuli. In general, VECs show significant metabolic changes during activation, resulting in further VEC dysfunction and development of cardiovascular disease, diabetic angiopathy, and tumor angiogenesis. For example, the activation and dysfunction of VECs in vulnerable areas of arterial blood vessels can be easily detected in the development of atherosclerosis (Davignon and Ganz, 2004), accompanied by unique metabolic reprogramming of VECs (Goveia et al., 2014). Moreover, vascular dysfunction caused by VEC metabolic changes initiates the development of diabetic angiopathy and contributes to the pathogenesis of vascular complications (Shi and Vanhoutte, 2017). In addition to vascular growth factors, VEC metabolism also plays a significant role in tumor neovascularization (Rohlenova et al., 2018). The abovementioned studies indicate that VEC metabolism might be a possible therapeutic target of vasculature related diseases.

At present, one of the important strategies targeting VEC metabolism is the development of anti-angiogenic drugs (Lopes-Coelho et al., 2020). PFKFB3 is an important enzyme in glycolysis, and participates in the synthesis of ATP and biological macromolecules. Initial study has demonstrated that the inhibition of the gene *PFKFB3* may reduce the formation of vessels under physiological condition (De Bock et al., 2013b). Notably, anti-glycolysis therapy with the PFKFB3 blocker 3-(3-pyridinyl)-1-(4-pyridinyl)-2-propen-1-one(3PO) can reduce glycolysis and pathological angiogenesis (Schoors et al., 2014), and a phase 1 clinical trial of the 3PO derivative PFK158 for the treatment of solid malignant tumor has been carried out (Li et al., 2019b). Therapy targeting PFKFB3 is safe because it temporarily inhibits only a branch of glycolysis and does not harm other healthy tissues that depend on glycolysis for their energy production (Schoors et al., 2014). Besides, Schoonjans et al. have demonstrated that PDK inhibitor dichloroacetate (DCA) and GLS1 inhibitor Bis-2-(5-phenylacetamido-1,3,4-thiadiazol-2-yl) ethyl sulfide (BPTES) affect tumor angiogenesis by lowering glycolysis and glutamate production, respectively, in HUVECs (Schoonjans et al., 2020). The silence of CPT1 in VECs can lead to the proliferation of VECs. As a result, it suggests that the pharmacological blockade of CPT1, such as etomoxir, may be used for the treatment of pathological angiogenesis by lowering FAO (Schoors et al., 2015). Further strategies to recover abnormal metabolism, decrease ROS production, and enhance the clearance of ROS, seem to be able to help counteracting diabetic vascular disease initiated by VEC dysfunction. However, clinical antioxidants have not been successfully applied to treat diabetic complication, to some extent (Ceriello et al., 2016). Therefore, new therapeutic targets are a continued focus of research related to vascular complications in diabetes. Nonetheless, VEC-based metabolic targeted therapies for atherosclerosis had not been developed to date.

As our understanding of VEC metabolism improves, new treatment options may continue to emerge. The detailed characterization of VEC metabolism under normal and pathophysiological conditions, direct metabolic flux measurements, and more comprehensive metabolomics analyses may provide future treatment targets for a wide range of pathologies related to abnormal vascular development.

## Author Contributions

HP sorted out, reviewed, and analyzed the literatures, drew the diagrams, and wrote the manuscript. XW sorted out, reviewed, and analyzed the literatures and revised the manuscript. HJ devised the concept. JD, QC, YH, and HJ supervised the writing. All authors revised and approved the final version of the manuscript.

## Conflict of Interest

The authors declare that the research was conducted in the absence of any commercial or financial relationships that could be construed as a potential conflict of interest.
